# Environmental Design Shapes Perceptual-motor Exploration, Learning, and Transfer in Climbing

**DOI:** 10.3389/fpsyg.2015.01819

**Published:** 2015-11-25

**Authors:** Ludovic Seifert, Jérémie Boulanger, Dominic Orth, Keith Davids

**Affiliations:** ^1^CETAPS - EA 3832, Faculty of Sport Sciences, University of RouenRouen, France; ^2^Laboratoire CRISTAL, UMR 9189, University of Lille 1Lille, France; ^3^School of Exercise and Nutrition Science, Queensland University of TechnologyBrisbane, QLD Australia; ^4^Centre for Sports Engineering Research, Sheffield Hallam UniversitySheffield, UK

**Keywords:** coordination dynamics, motor learning, meta-stability, exploration, affordances

## Abstract

This study investigated how environmental design shapes perceptual-motor exploration, when meta-stable regions of performance are created. Here, we examined how creating meta-stable regions of performance could destabilize pre-existing skills, favoring greater exploration of performance environments, exemplified in this study by climbing surfaces. In this investigation we manipulated hold orientations on an indoor climbing wall to examine how nine climbers explored, learned, and transferred various trunk-rolling motion patterns and hand grasping movements. The learning protocol consisted of four sessions, in which climbers randomly ascended three different routes, as fluently as possible. All three routes were 10.3 m in height and composed of 20 hand-holds at the same locations on an artificial climbing wall; only hold orientations were altered: (i) a horizontal-edge route was designed to afford horizontal hold grasping, (ii) a vertical-edge route afforded vertical hold grasping, and (iii), a double-edge route was designed to afford both horizontal and vertical hold grasping. As a meta-stable condition of performance invite an individual to both exploit his pre-existing behavioral repertoire (i.e., horizontal hold grasping pattern and trunk face to the wall) and explore new behaviors (i.e., vertical hold grasping and trunk side to the wall), it was hypothesized that the double-edge route characterized a meta-stable region of performance. Data were collected from inertial measurement units located on the neck and hip of each climber, allowing us to compute rolling motion referenced to the artificial climbing wall. Information on ascent duration, the number of exploratory and performatory movements for locating hand-holds, and hip path was also observed in video footage from a frontal camera worn by participants. Climbing fluency was assessed by calculating geometric index of entropy. Results showed that the meta-stable condition of performance may have afforded utilization of more adaptive climbing behaviors (expressed in higher values for range and variability of trunk rolling motion and greater number of exploratory movements). Findings indicated how climbers learn to explore and, subsequently, use effective exploratory search strategies that can facilitate transfer of learning to performance in novel climbing environments.

## Introduction

Learning new skills result in relatively permanent changes in an individual's behaviors due to learning experiences under a specific set of performance constraints (Newell, 1996). In the theoretical framework of ecological dynamics, the problem of learning a new skill is conceptualized as a reorganization of a behavioral repertoire (Schöner et al., 1992; Zanone and Kelso, 1992; Davids et al., 2012). In other words, it is postulated that learning a new skill would require destabilization of pre-existing skills, leading to a reorganization of a behavioral repertoire (Schöner et al., 1992; Faugloire et al., 2009), leading to skill “adaptation” (Araújo and Davids, 2011). Adaptation of existing behaviors to a new performance context involves the process of skill transfer (Newell, 1996; Zanone and Kelso, 1997; Kelso and Zanone, 2002).

Previous research supporting a destabilization hypothesis has emerged from studies of bimanual coordination and postural regulation, where relationships between learning and pre-existing behaviors have been investigated when to-be-learned skills have been instructed (i.e., when an informational constraint is imposed). A crucial question concerns the relationships between learning and pre-existing skills when to-be-learned skills are induced through exploratory activity (Nourrit et al., 2003; Teulier and Delignières, 2007; Chow et al., 2008) and transfer (Zanone and Kelso, 1997; Kelso and Zanone, 2002) processes that emerge when task and/or environmental constraints are manipulated. Skill transfer occurs when prior experiences under a particular set of constraints influences performance behaviors in a different set of constraints compared to those where the skills were originally learned (Newell, 1996; Rosalie and Müller, 2012). For this reason, practice task constraints should seek to simulate aspects of ecological constraints of performance environments. Pre-existing skills serve as a foundation that a learner can use to identify new coordination patterns and information-movement couplings (Newell, 1991, 1996; Zanone and Kelso, 1997; Davids et al., 2008). In particular, capacity to transfer learning to different performance conditions has been related to opportunities to explore different coordination tendencies and the information-movement couplings that regulate them (Newell et al., 1989, 1991; Newell and McDonald, 1992). It seems that the nature of existing intrinsic dynamics, and the current task-goal, underpins the level of transfer (i.e., exploitation of the pre-existing skills) and exploration (i.e., emergence of a new skill) needed in motor learning.

Exploration of a perceptual-motor workspace when learning a new skill has been extensively investigated previously (Newell, 1985, 1991; Newell et al., 1989, 1991). The “perceptual-motor workspace” represents all the hypothetical possible coordination patterns available that learners can explore during goal-directed activity (Newell et al., 1991, 1989). To enhance the learning process, a perceptual-motor workspace can be constrained (e.g., at task, organismic, environmental levels; Newell, 1986) in order to set boundaries for exploration in a “field of promoted action” according to Reed and Bril (1996). Reed and Bril (1996) defined a field of promoted action as an environment where an experimenter can introduce information in the form of objects, places and activities, designed to influence the information-movement couplings that are used to seek and utilize behavioral opportunities termed *affordances* (Gibson, 1979). Affordances are relationships between the structural (e.g., limb size) and functional (e.g., movement pattern) aspects of an individual relative to physical properties in the environment (Gibson, 1979). Thus, a field of promoted action can be designed where an action may be more or less encouraged or discouraged, when certain artifacts and some affordances may be more or less widely available in an individual's environment (Reed and Bril, 1996). Affordances invite ways of achieving behavioral goals (Withagen et al., 2012) and when multiple affordances are made to overlap by manipulating constraints, exploratory behaviors emerge in an intermittent or meta-stable regime of performance (Hristovski et al., 2006b, 2009; Pinder et al., 2012).

Meta-stability in complex dynamical systems has been conceptualized as a region of performance where competing tendencies simultaneously coexist: the tendency of system components to couple together and the tendency for components to express their intrinsic independent behaviors (Tognoli and Kelso, 2009; Kelso, 2012). During performance, components of a movement system and a performance environment become temporarily coupled. Coordination of these couplings in the meta-stable regime is conceptualized as being weakly attracted toward multiple states of stability without an actual fixed state of stability being present (Kelso, 2012). In other words, in a meta-stable region, there is no stable pattern of coordination, however the initial attraction to what the attractors were still persists Kelso (2008), leading Kelso and Engström (2006) to say that, “there is attractiveness but, strictly speaking, no attractor” (p. 172). This type of system flexibility supports the ability to transition among different coordination tendencies to adapt to changes in internal and external constraints on performance and find more functional performance solutions (Tognoli and Kelso, 2009; Kelso, 2012). Meta-stability refers to a regime that is neither stable nor totally un-stable (Kelso, 2012), where learners can depart from their initial stable behaviors to explore new opportunities, providing the scaffolding for exploratory behaviors during motor learning (Sporns and Edelman, 1993). Exploration through a meta-stable regime may occur more or less abruptly and over a more or less extended period of time with possibility of fall back transitions between the initial behaviors and emergent possibilities until a new stable coordination tendency is stabilized (Teulier and Delignières, 2007). For instance, a recent study in soccer kicking, where participants had to kick a ball over a horizontal barrier to reach a target placed at various distances, demonstrated that learners explored different coordination patterns with turnovers between those patterns (Chow et al., 2008). Indeed, an initial novice coordination pattern was used as a platform to transit toward more skilled behaviors. At the same time the emerging behavior remained only relatively stable to allow participants to fall back toward the original behaviors if needed (Teulier and Delignières, 2007). Such progressive and regressive exploration of a perceptual motor workspace characterizes motor learning in complex, adaptive systems (Newell and Vaillancourt, 2001) offering a secure opportunity to explore novel behaviors and also the opportunity for skill transfer.

Based on these theoretical insights, an interesting research question concerns how to design a meta-stable regime of performance, to encourage exploration of a perceptual-motor workspace. Observations of performance within meta-stable regimes has suggested that numerous coordination patterns can spontaneously emerge under interacting constraints (see Hristovski et al., 2006a,b), allowing individuals to vary their goal-directed behaviors during performance (Pinder et al., 2012). Meta-stability has been tested in studies involving complex multi-articular actions such as striking a heavy bag in boxing (Hristovski et al., 2009) and cricket batting (Pinder et al., 2012). These studies showed that under a meta-stable performance regime (respectively, the distance between boxers' and a heavy bag to-be-intercepted, and the distance between cricket batters and the bounce of a ball to be hit) individuals varied trial-to-trial movement patterns to achieve the task-goal. These studies showed how, during periods of meta-stability, complex, adaptive movement systems can functionally reorganize actions from which new functional behaviors can emerge. However, the issue of designing meta-stable regions for performance of multi-articular actions during learning remains untested. A pilot study showed that the meta-stable region of performance in a climbing task (i.e., a “crux” point) induced a higher number of varied behaviors (hold grasping patterns), a higher number of falls and a higher number of exploratory actions (e.g., touched holds), which all decreased with extended practice (Seifert et al., 2013b). These initial data implied that the functionality of behavioral variability induced by a meta-stable region of performance (and that continued to be present across practice) may have facilitated the emergence of more adaptive climbing actions.

Therefore, the aim of our study was to investigate the role of constraints manipulation (i.e., opportunity of actions offered by the holds on an indoor climbing wall) in inducing meta-stability in learners acquiring skill in a complex multi-articular action in a challenging environment (i.e., a climbing task). Specifically, we sought to examine how individuals learned and transferred various trunk-rolling motion patterns and hand grasping movements in a climbing task when hold orientation was manipulated to induce meta-stability. Our first hypothesis was that meta-stable region of performance would invite an individual to both exploit his pre-existing behavioral repertoire (i.e., horizontal hold grasping pattern and trunk face to the wall) and explore new behaviors (i.e., vertical hold grasping and trunk side to the wall). Therefore, by favoring greater exploration of the hand grasping movements (e.g., in terms of *touched* vs. *grasped* hand-holds; see Pijpers et al., 2006) and of trunk-rolling motion patterns, meta-stable region of performance would destabilize pre-existing behaviors and lead to a reorganization of the behavioral repertoire. We also hypothesized that practice in a meta-stable region of performance may lead participants to learn how to explore. We expected that there would be a consequent effect on behavioral exploration that can be transferred to a novel performance situation.

## Methods

### Participants

Nine male participants voluntary participated in this study, with a mean age of 21.9 ± 2.7 years; mean height: 178.4 ± 5.8 cm; mean weight: 72.1 ± 5.8 kg. Participants had practiced indoor climbing for 3 h per week for 3 years. They had a skill level in rock climbing of grade 6a-6b on the French Rating Scale of Difficulty (F-RSD) (ranging from 1 to 9) (Delignières et al., 1993), which represents an intermediate level of performance (Draper et al., 2011) corresponding to the control stage of motor learning (Newell, 1985).

### Protocol

**Figure 1:**
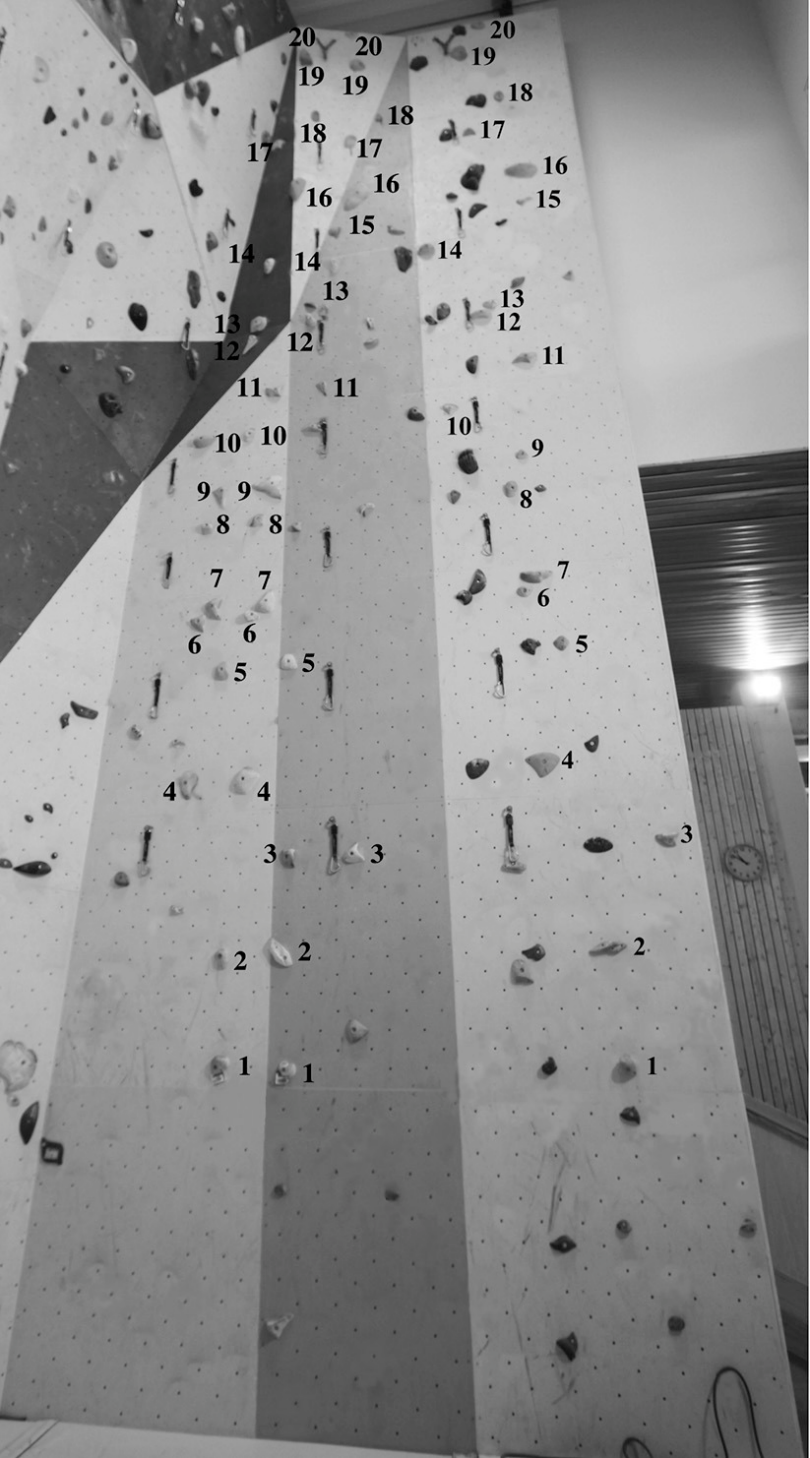
**Location of the 20 hand-holds for the three routes**.

The learning protocol consisted of four sessions in the climbing task (each separated by 2 days of rest), where participants were instructed to self-pace their ascent. Each session consisted of ascending randomly three different routes of a grade ranged 5b-5c on the F-RSD. Therefore, the designated climbing task corresponded to a sub-maximal grade of difficulty for the participants, which prevented any possible fatigue effects during each testing session. They had the following task-goal: to find the way to climb up the surface as *fluently* possible, i.e., without falling down and by minimizing pauses and rest periods of body displacement during the ascent (Cordier et al., 1993, 1994; Seifert et al., 2014b). Instructions were not made too specific to allow coordination patterns to emerge as each climber exhibited exploratory behaviors under the varying task constraints. Each route was identifiable by different color and was set on an artificial indoor climbing wall by three professionally-certified route setters who ensured that the routes matched an intermediate climbing ability. The three routes had the same height (10.3 m) and they were composed of the same number of hand-holds (20), which were bolted to a flat vertical surface (Figure 1).

**Figure 2:**
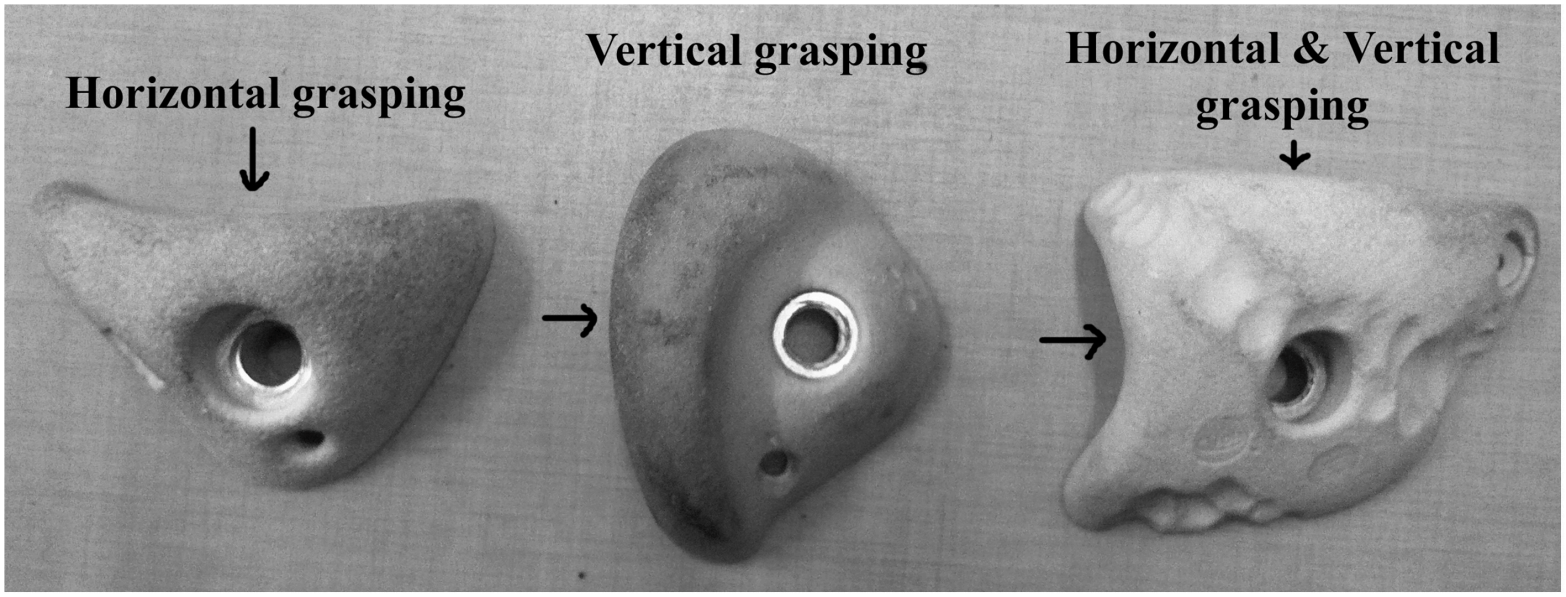
**Orientation and shape of the holds for the three routes**. The arrow indicates the preferential grasping allowed by the hold.

The distance between holds was kept constant for these three routes; only the orientation of the hold was changed: (i) a horizontal-edge route was designed to allow horizontal hold grasping, (ii) a vertical-edge route was designed to allow vertical hold grasping, and (iii), a double-edge route was designed to allow both horizontal and vertical hold grasping. In the latter, each hold had two edges: a horizontal edge that could be grasped with the knuckles in-line running parallel to the horizontal axis, and a vertical edge that could be grasped with the knuckles parallel to the vertical axis (Figure 2). Each edge could also be grasped by the left and/or the right hand. According to a pilot study by Seifert et al. (2013b), the horizontal-edge route led to the emergence of a horizontal hold grasping pattern with the trunk face to the wall, like “climbing a ladder,” which was assumed to be a *cooperative* situation where individuals *exploited* their pre-existing behavioral repertoires. The vertical-edge route led to a vertical hold grasping pattern and the rolling motion of the trunk to the side of or obliquely to the wall, like “opening/closing a door.” This was assumed to be a more *competitive* situation relative to the pre-existing behavioral repertoire, where individuals *explored* new behaviors. It was hypothesized that the double-edge route could be characterized as a meta-stable condition of performance. This was because an individual could both *exploit* the pre-existing behavioral repertoire (i.e., horizontal hold grasping pattern and trunk face to the wall) and *explore* new behaviors (i.e., vertical hold grasping pattern and rolling motion of the trunk side or obliquely to the wall), which can finally be observed by greater movement and coordination patterns variability.

Each route was top-roped, i.e., the route was climbed with a rope anchored above the climber at all times (while lead climbing is where the climber clips the rope into intermediate anchors along the climbing route). Each ascent was preceded by 3 min of route previewing, since a pre-ascent climbing route visual inspection was deemed to be a key parameter of successful climbing performance (Sanchez et al., 2012).

In the fourth session, the participants climbed a fourth different route, for which the design was a mixture of the three previous routes: the first six holds only allowed horizontal grasping, then the next seven holds only allowed vertical grasping, while the last seven holds allowed horizontal and vertical grasping. This new route was designed to assess the capability of the climbers to reinvest the grasping patterns they had learned in the three other routes and corresponded to a transfer test.

This study was carried out in accordance with the recommendations of the guidelines of the International Committee of Medical Journal Editors with written informed consent from all participants. A local University ethics committee approved the protocol. The protocol was explained to all participants who gave written informed consent in accordance with the Declaration of Helsinki. Vulnerable populations (e.g., minors, persons with disabilities) were not involved in the study.

### Data collection and analysis

Data on ascent duration, the number of exploratory (touched holds) and performatory (grasped holds) movements on hand-holds (Pijpers et al., 2006), the hip path, and for the double-edge route, the type of hand grasping patterns (vertical vs. horizontal-edge grasping) were collected from video footage. This occurred with a frontal camera (Sony EX-View Super HAD, with a 2.6 mm lens that offered a 120° angle of view) fixed 9.5 m away from the climbing wall and at a distance of 5.4 m from the ground. A calibration frame, 10.3 m vertical × 3 m horizontal and composed by 20 markers, was used to correct for parallax, distortion, and calibrate the digitized trajectory of the hip from pixels to meters by using Matlab® R2014a (1994–2014, The MathWorks, Inc.). As the task-goal of the participants was to climb with highest fluency, the climbing fluency of the hip trajectory was assessed by the geometric index of entropy (GIE) (Cordier et al., 1993, 1994; Sibella et al., 2007). GIE was calculated by recording the distance path covered by the hip (L) and the perimeter of the convex hull around that path (c) according to the following equation (Cordier et al., 1993, 1994): GIE = logn2L/c. According to Cordier et al. (1993, 1994), the geometric index of entropy measures reveal the amount of fluency/curvature of a curve. The higher the entropy, the higher the disorder of the system: therefore, a low entropy value was associated with low energy expenditure and greater climbing fluency. Clearly, an excessive deviation of the hip trajectory from the vertical axis, which could be due to route finding and hold exploration may compromise climbing fluency (Cordier et al., 1993, 1994; Seifert et al., 2014b), so that the number of exploratory and performatory movements can indicate the capacity of climbers to pick up functional features of the climbing wall, and to improve information-movement couplings with practice. Exploratory and performatory movements are defined by Pijpers et al. (2006) according to whether a potential hold on a climbing wall was touched, with or without it being used as support.

**Figure 3:**
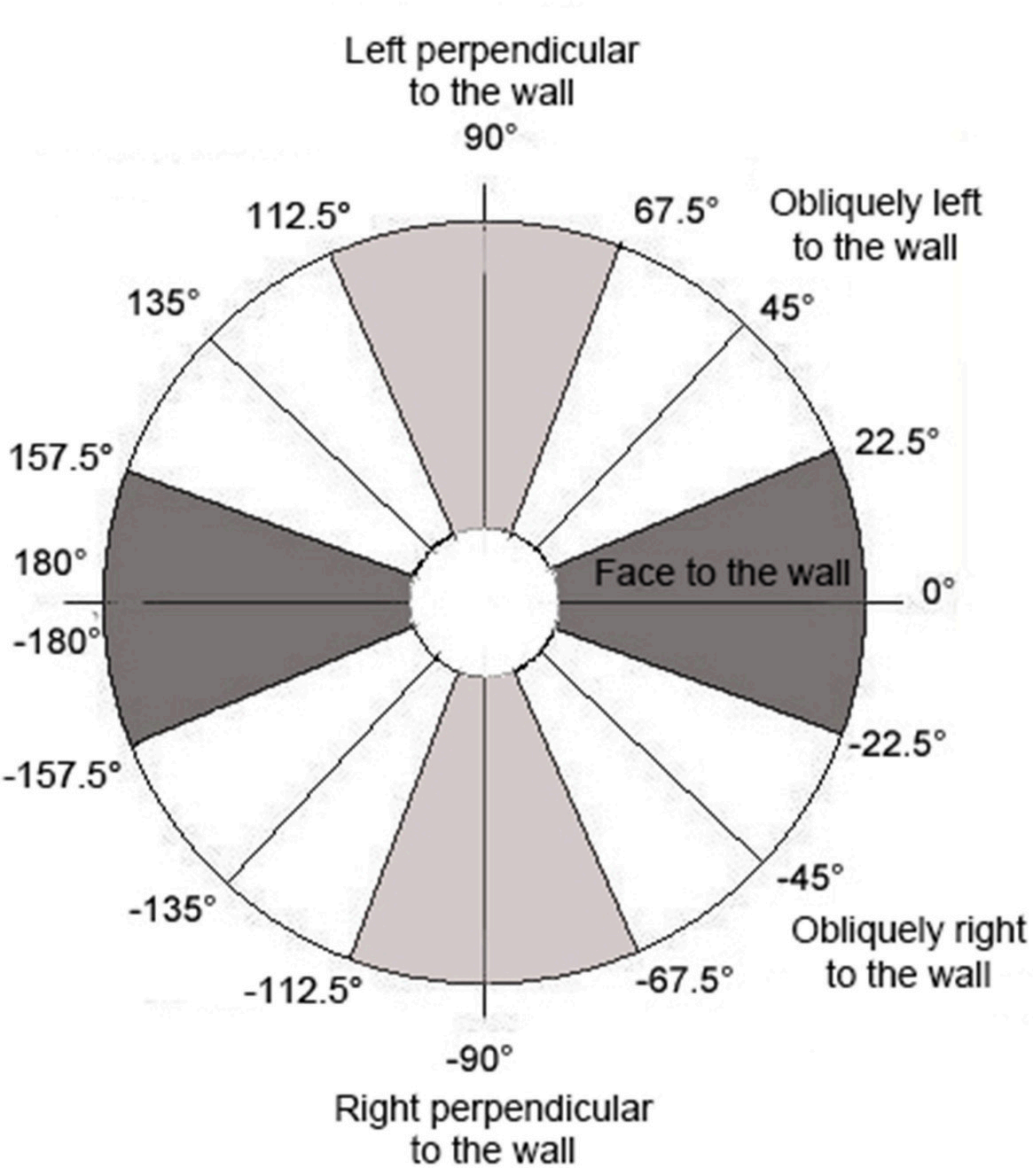
**Rolling motion patterns of the ***neck*** and ***hip*** according to climbing wall reference**.

Moreover, exploratory behavior is not limited to limb actions but also concerns trunk translation and oscillation (Cordier et al., 1996; Seifert et al., 2014b). Previous studies have attempted to compute climbing skills from measures of head (Pansiot et al., 2008) or wrist motion (Ladha et al., 2013) using a single accelerometer sensor. The findings were severely limited due to the inability to record acceleration using an Earth reference frame (magnetic North, East, and gravity directions). To overcome this problem, we collected data on trunk direction (3D unitary vector in Earth reference) from small wearable inertial measurement units (IMU) located on the neck and hip of climbers, with a magnetic North reference, sampled at 100 Hz. A third IMU was also placed on the artificial climbing wall to locate it on the Earth reference frame. Our IMUs corresponded to a combination of a tri-axial accelerometer (±8G), tri-axial gyroscope (1600°.s^−1^) and a tri-axial magnetometer (*MotionPod*, Movea^©^, Grenoble, France) and have been already used to assess the measure of jerk of the hip trajectory during a climbing task (Seifert et al., 2014b), and limb 3D direction (Seifert et al., 2014a). Wireless transmissions to a controller enabled recording by *MotionDevTool* software (Movea^©^, Grenoble, France). The neck, hip and climbing wall orientation (a 3D frame, constituted by three 3D unit vectors) were computed with respect to the Earth reference by using a complementary filter-based algorithm, which integrated the three sensor information sources (i.e., accelerometer, gyroscope, and magnetometer) (Madgwick, 2010; Madgwick et al., 2011). Then, the angular time series corresponding to the neck and hip rolling motion around the vertical axis were extracted according to the climbing wall reference: 0° of roll corresponded to face-to-the wall position; positive angle values of roll corresponded to left side-to-the wall positions (e.g., 90° corresponded to left perpendicular trunk position to the wall); negative angle values of roll corresponded to right side-to-the wall positions (e.g., −90° corresponded to right perpendicular trunk position to the wall). As in previous research assessing upper and lower-limb angular positioning in ice climbing (Seifert et al., 2013c, 2014c), relative time (expressed in % of ascent duration) spent in face vs. side positions to the wall was computed by distinguishing five main patterns of rolling motion: face (−22.5° to 22.5°), obliquely right (−22.6° to −67.5°), obliquely left (22.6° to 67.5°), right perpendicular (−67.6° to −112.5°) and left perpendicular (67.5° to 112.5°) orientations to the wall (Figure 3). Although, these five patterns did not represent the full suite of angular possibilities, none of the collected data had an angular roll larger in absolute value than 112.5°.

The coordination between neck and hip rolling motions was computed with a local correlation coefficient. This signified that the correlation values were not computed on the whole signal but solely in a sliding window of 5 s (a window of 5 s was selected because it corresponds to the average time used to perform one action successively with the: right hand, left hand, right foot, and left foot). This procedure enabled the synchronized information embedded in neck and hip rolling motions on a local scale to be retrieved since such synchronized information evolved during the trial and could not be summed with just a single measure. Local correlation coefficients were computed using the following formula (Mardia and Jupp, 1999):

$$C(t)=\frac{cos\;(S_{1}(t:t\prime ),\;S_{2}(t:t\prime ))}{\sqrt{\begin{array}{l} cov\;(S_{1}\;(t:t\prime ),\;S_{1}\;(t:t\prime ))\\ cov\;(S_{2}(t:t\prime ),\;S_{2}(t:t\prime )) \end{array}}}$$

$$\begin{array}{l} \text{where }cov(S_{1},S_{2}) =\bar{cos(S_{1})cos(S_{2})}*\bar{sin(S_{1})sin(S_{2})}\\ \;-\;\bar{cos(S_{1})sin(S_{2})}*\bar{sin(S_{1})cos(S_{2})} \end{array}$$

and where *C* is the local correlation, *S1* and *S2* are the signals for neck and hip, *t*′ equals *t* + *w*, where *w* the sliding window duration in seconds (5 s in this study). *S*(*t* : *t*′) denotes the signal S taken from time *t* to time *t*′. As in previous research (e.g., Chow et al., 2014), three thresholds of coefficient correlation were selected to quantify the nature of neck-hip coordination: (i) an in-phase pattern was deemed to be present if the local correlation coefficient was more than 0.25, meaning that the trunk is rolling in one block; (ii) a no-phase pattern was present if the local correlation coefficient was between −0.25 and 0.25; (iii) an anti-phase pattern was present if the local correlation coefficient was less than −0.25, meaning that the neck and the hip are twisting (Amblard et al., 1994; Temprado et al., 1997 on the rationale for such a selection).

As in previous motor learning study (Kostrubiec et al., 2012), the angular mean (μ) and the angular concentration (*K*) were estimated for each time series, computed as the maximum likelihood estimator of the Von Mises distribution (Mardia and Jupp, 1999):

$$f\left(s|\mu ,\kappa \right)=\frac{e^{\kappa \;cos\left(s-\mu \right)}}{2\pi I_{0}\left(\kappa \right)}$$

with *I*_0_ the modified Bessel function of order 0.

The angular mean (μ) represents the average angle of the time series while the concentration (*K*) can be interpreted as the inverse of the variance from a classical normal random variable. If *K* = 0, the angular data are uniform, if *K* is large, the angular time series is concentrated around μ.

### Statistical analysis

Effects of practice and route design were analyzed by separate Two-way repeated measures ANOVAs (practice across four sessions and climbing wall design across three different routes) for neck and hip rolling motion, neck-hip coordination, number of exploratory and performatory movements, ascent duration and geometric index of entropy. Sphericity in the repeated measures design was verified with the Mauchly test (Winter et al., 2001). When the assumption of sphericity was not met, the significance levels of *F*-ratios were adjusted according to the Greenhouse-Geisser procedure. *Post-hoc* pairwise condition comparison tests were applied and family-wise error rate was controlled by applying a Bonferroni correction of the *P*-value (Howell, 2002).

Finally, skill transfer was analyzed for the same dependent variables using One-way repeated measures ANOVA and simple contrast tests. Simple contrast tests were used to examine how practice on *known routes* affected performance on *new routes*, by comparing the fourth session of each route with the transfer test. Since a great part of climbing skills corresponds to *on-sight* climbing (i.e., with *no prior knowledge of the route*), simple contrast tests were also used to investigate the effect of practice on climbing unknown routes, by comparing the first session of each route with the transfer test. These two contrast tests are interesting because practice can have an effect on route finding. Therefore, it was hypothesized that first session of each route and the transfer test would reveal differences in climbing fluency, but similar exploratory behavior. However, we expected positive effects of exploration, meaning that no significant differences in climbing fluency and lower exploratory behavior were hypothesized to emerge between the fourth session and a transfer test. Partial eta squared ($\eta_{\text{P}}^{2}$) statistics were calculated as an indicator of effect size, considering that $\eta_{\text{P}}^{2}=0.01$ represents a small effect, $\eta_{\text{P}}^{2}=0.06$ represents a medium effect and $\eta_{\text{P}}^{2}=0.15$ represents a large effect (Cohen, 1988). All tests were performed using IBM SPSS Statistics 20.0 (1989–2011), with a level of statistical significance fixed at *p* < 0.05.

## Results

**Figure 4:**
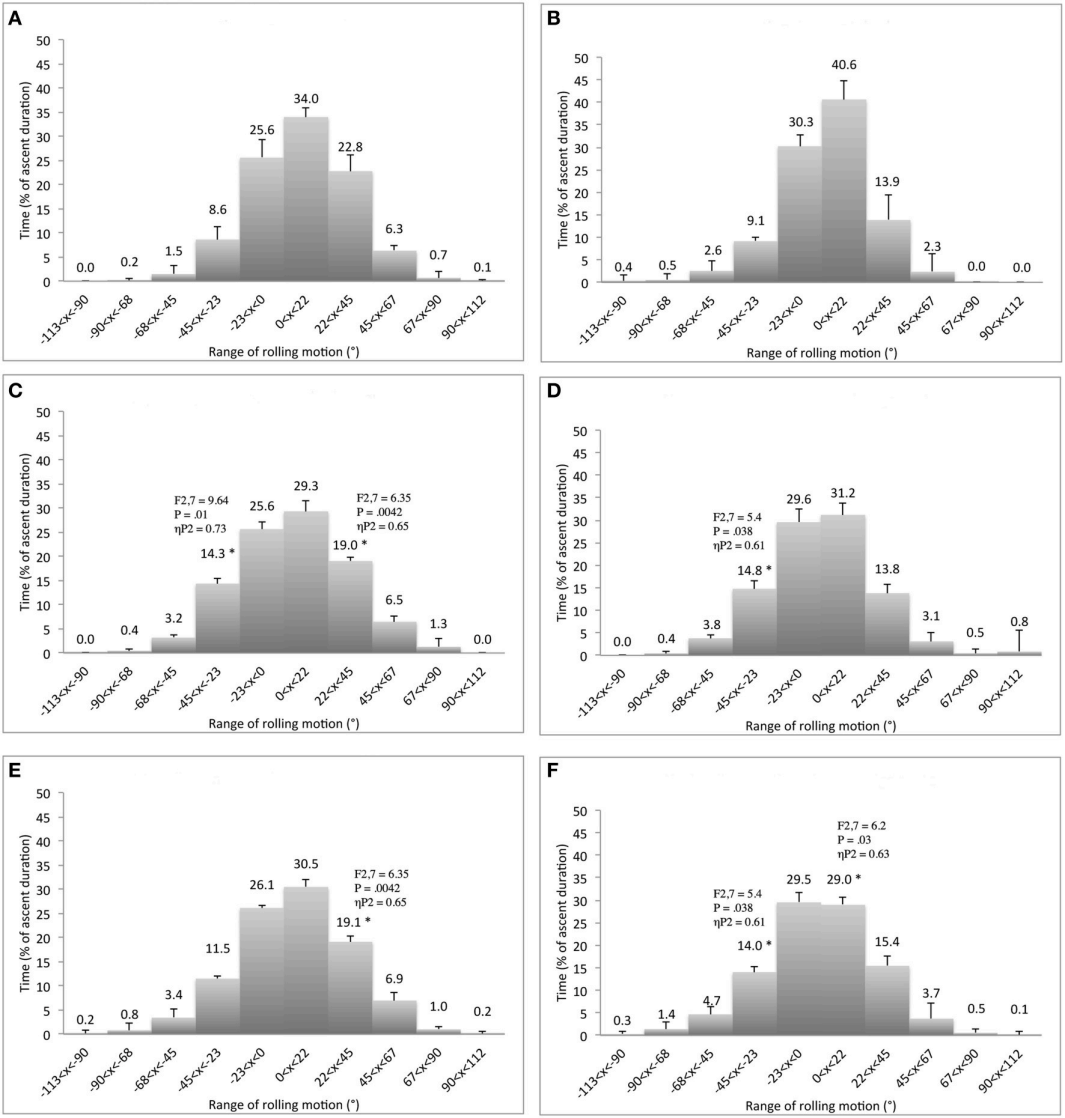
**Differences of relative duration spent to roll the ***hip*** (left panels) and ***neck*** (right panels) between the horizontal-edge (A,B), vertical-edge (C,D), and double-edge routes (E,F)**. ^*^Corresponds to significant differences with the horizontal-edge route (including *F*- and *P*-values and effect size: $\eta_{\text{P}}^{2}$).

Overall (when the three routes and the four learning sessions are considered), the climbers spent 55–75% of the time with the trunk in a *face* to wall orientation (Figure 4). The rest of the time was mainly spent with the trunk in an *oblique* position, while less than 2% of the time was spent with the trunk *side* to the wall (Figure 4). This rolling motion of the trunk was achieved by using an in-phase pattern of neck-hip coordination 65–85% of the time, with an anti-phase pattern used 10–20% of the time. However, *route design* and *practice* showed significant behavioral adaptations that were also used during the transfer test. These results are presented next in three sections.

### Effect of route design

The *route design* had a significant effect on the hip and neck rolling motion *variability*, but not on the coordination of the neck-hip rolling motions. In particular, the horizontal-edge routes (for hip, *K* = 9.2; for neck, *K* = 12.5 variables) exhibited significantly higher concentration of the distribution than the vertical-edge route (for hip, *K* = 6.3; for neck, *K* = 7.8) and the double-edge route (for hip, *K* = 6.1; for neck, *K* = 6.6) [for hip, *F*_(1.171, 9.369)_ = 8.26, *p* = 0.015, $\eta_{\text{P}}^{2}=0.51$ and for neck *F*_(1.152, 9.218)_ = 17.37, *p* = 0.002, $\eta_{\text{P}}^{2}=0.69$]. Deeper analysis of route design showed that the horizontal-edge route led the climbers to roll their trunk *obliquely* to the wall for significantly shorter durations than the vertical-edge and/or the double-edge routes (Figure 4). According to Figure 4, the effect of route design was more pronounced at the level of the neck level than the hip, which was directly linked to the type of hand grasping patterns used by participants because hold orientation influenced hand grasping patterns that influences shoulder rolling motions. Since we assumed that the vertical-edge route only allowed vertical hand grasping patterns, while the 20 holds of the double-edge route could be grasped vertically or horizontally, the hand grasping patterns used by participants were recorded for each ascent performed on the double-edge route. Results averaged over the four learning sessions revealed that 9 of the 22 hand grasping movements performed on the double-edge route were with the vertical-edge grasping pattern. Moreover, the horizontal-edge route resulted in significantly fewer exploratory and performatory movements, lower times for ascent duration and a smaller geometric index of entropy (i.e., higher climbing fluency), than the vertical-edge and/or the double-edge routes (Table 1).

**Table 1:** **Differences of performatory movements, exploratory movements, ascent duration and geometric index of entropy between the horizontal-edge, vertical-edge, and double-edge routes**.

	**Performatory movements**		**Exploratory movements**		**Ascent duration (s)**		**Geometric index of entropy**	
	**Mean**	***SD***	**Mean**	***SD***	**Mean**	***SD***	**Mean**	***SD***
Horizontal-edge route	20.7	0.4	0.2	0.1	74.6	9.0	1.08	0.1
Vertical-edge route	22.3^*^	0.4	0.6^*^	0.1	114.2^*^	16.2	1.14	0.2
Double-edge route	22.2^*^	0.4	0.5^*^	0.1	118.1^*^	15.7	1.21^*^	0.2
*F*-value	*F*_(2, 7)_ = 11.45		*F*_(2, 7)_ = 5.66		*F*_(2, 7)_ = 10.21		*F*_(2, 7)_ = 8.8	
*P*-value	*P* = 0.006		*P* = 0.035		*P* = 0.008		*P* = 0.012	
Effect size	$\eta_{\text{p}}^{2}=0.77$		$\eta_{\text{p}}^{2}=0.62$		$\eta_{\text{p}}^{2}=0.74$		$\eta_{\text{p}}^{2}=0.72$	

* *Corresponds to significant differences with the horizontal-edge route with P < 0.05*.

### Effect of practice

**Figure 5:**
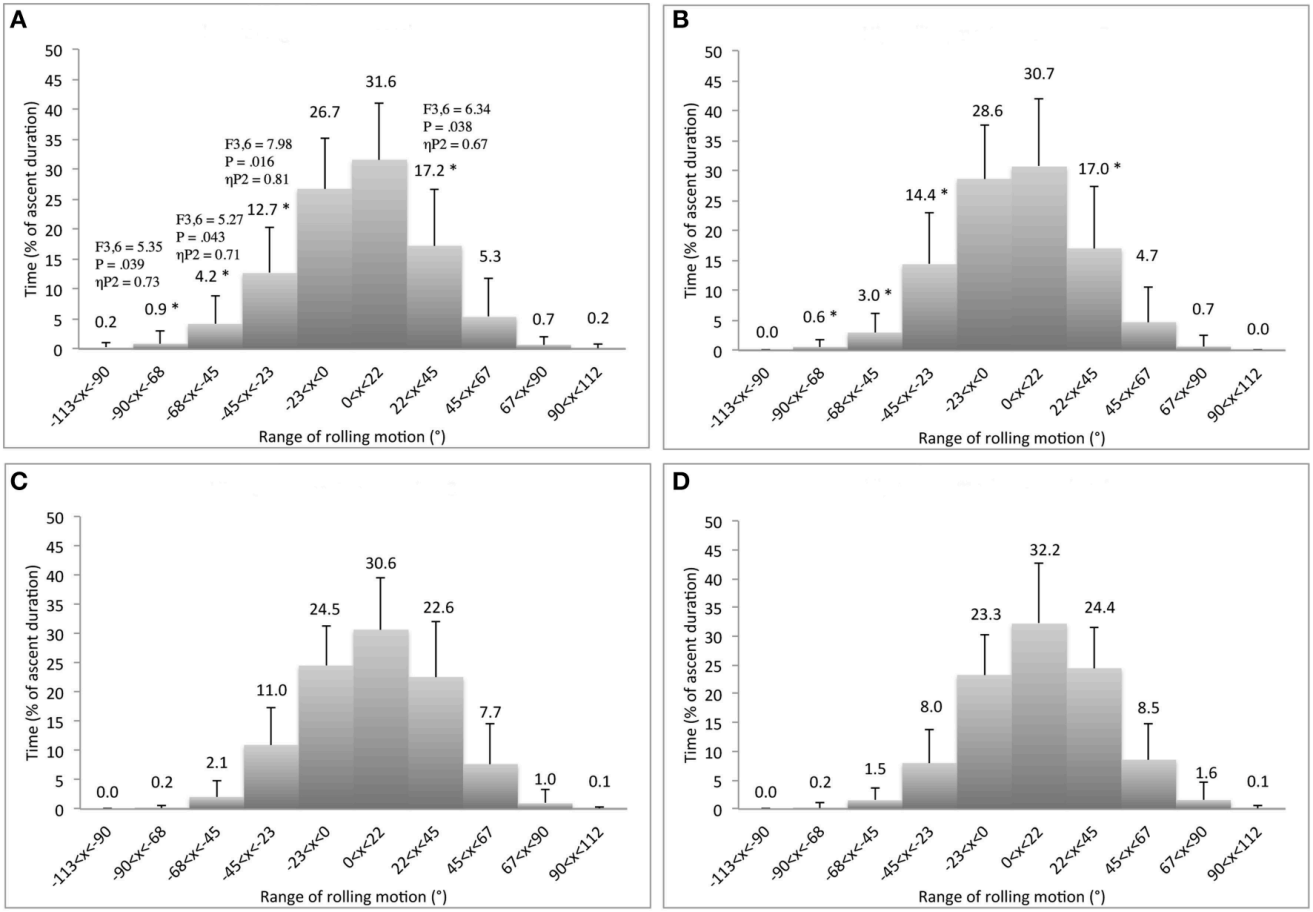
**Differences of relative duration spent to roll the ***hip*** between the first (A), the second (B), the third (C), and the fourth (D) learning session**. ^*^Corresponds to significant differences with the fourth session (including *F*- and *P*-values and effect size: $\eta_{\text{P}}^{2}$).

*Practice* did not show any significant effects on *variability* of trunk rolling motion. Similarly, practice did not show any significant effects on neck rolling motions and on coordination of the neck-hip rolling motions. However, practice had an impact on the *side* of the hip rolling motion, since climbers decreased the relative time spent rolling to the *left side* and increased the relative time spent rolling to the *right side* for sessions 1 and 2 in comparison to session 4 (Figure 5). Moreover, practice led to significantly fewer exploratory and performatory movements being observed and lower times of ascent duration over sessions (Table 2), but no change in geometric index of entropy (i.e., climbing fluency). Also, the number of hand vertical grasping movements on the double-edge route did not change significantly with practice (between 8.7 ± 2.7 and 9.4 ± 2.9). However, significant interactions between route design and practice showed that the geometric index of entropy significantly decreased between the two first sessions (respectively, 1.33 ± 0.1 and 1.25 ± 0.1) and the fourth session (1.08 ± 0.1) for the double-edge route, *F*_(3, 6)_ = 36.7, *p* = 0.007, $\eta_{\text{P}}^{2}=0.98$. Together, these results imply that practicing under a meta-stable regime entailed the same level of behavioral exploration associated with an improvement in performance outcomes (i.e., greater climbing fluency), supporting the effectiveness of the exploration process.

**Table 2:** **Differences of performatory movements, exploratory movements, ascent duration, and geometric index of entropy between sessions**.

	**Performatory movements**		**Exploratory movements**		**Ascent duration (s)**		**Geometric index of entropy**	
	**Mean**	***SD***	**Mean**	***SD***	**Mean**	***SD***	**Mean**	***SD***
Session 1	22.2	0.2	0.4	0.1	115.9	9.0	1.17	0.1
Session 2	21.8^*^	0.4	0.3	0.1	103.2	18.4	1.16	0.2
Session 3	21.4^*^	0.4	0.3	0.1	96.5^*^	13.7	1.15	0.2
Session 4	21.4^*^	0.4	0.1^*^	0.1	93.4^*^	14.1	1.11	0.1
*F*-value	*F*_(3, 6)_ = 6.81		*F*_(3, 6)_ = 6.77		*F*_(3, 6)_ = 5.95			
*P*-value	*P* = 0.024		*P* = 0.026		*P* = 0.036			
Effect size	$\eta_{\text{p}}^{2}=0.77$		$\eta_{\text{p}}^{2}=0.74$		$\eta_{\text{p}}^{2}=0.71$			

* *Corresponds to significant differences with the first session with P < 0.05*.

### Transfer

**Figure 6:**
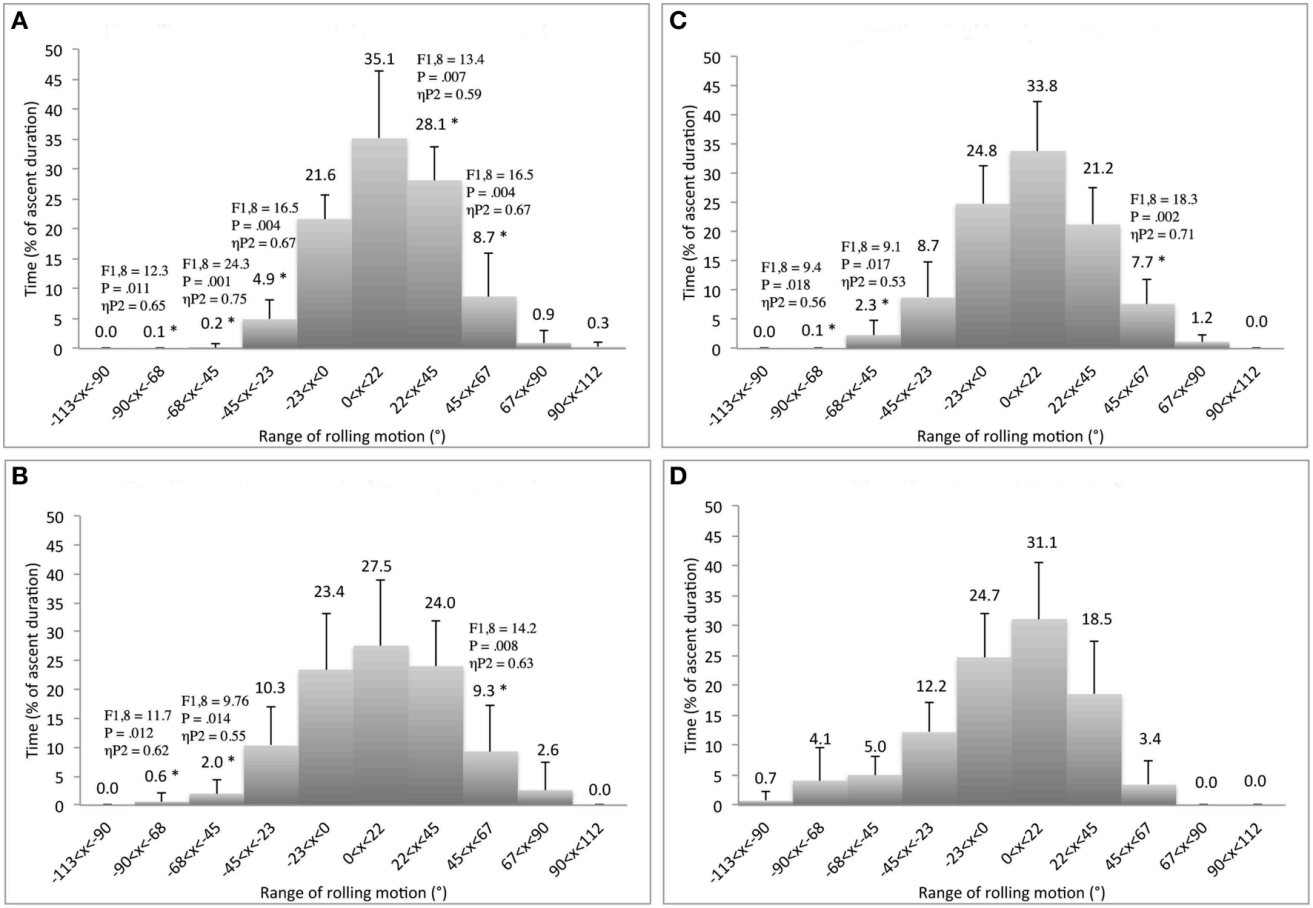
**Differences of relative duration spent to roll the ***hip*** between the ***fourth*** learning session of the horizontal-edge route (A), vertical-edge route (B), double-edge route (C), and the transfer test (D)**. ^*^Corresponds to significant differences with the transfer test (including *F*- and *P*-values and effect size: $\eta_{\text{P}}^{2}$).

**Figure 7:**
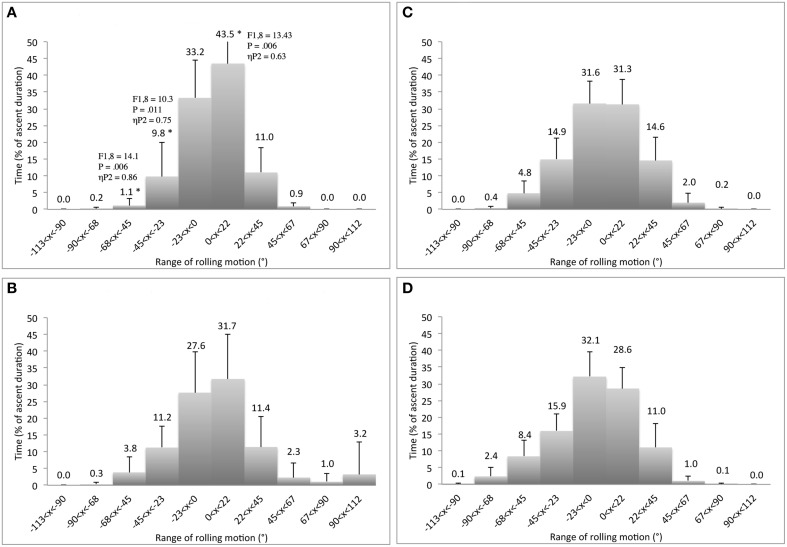
**Differences of relative duration spent to roll the ***neck*** between the ***fourth*** learning session of the horizontal-edge route (A), vertical-edge route (B), double-edge route (C), and the transfer test (D)**. ^*^Corresponds to significant differences with the transfer test (including *F*- and *P*-values and effect size: $\eta_{P}^{2}$).

When behavior during *known routes* (considering the fourth session of each route) was compared to that emerging during the transfer test, we noted higher levels of *variability* in trunk rolling motions, greater numbers of exploratory and performatory movements, and longer ascent duration times during the transfer test (Table 3). The higher level of exploratory behavior did not cause a decrease in climbing fluency because the climbers exhibited statistically non-significant differences in geometric index of entropy between the fourth session of each route and the transfer test, suggesting positive effects of exploration during learning. The higher *variability* in trunk rolling motions corresponded to the significant lower frequency of the *hip* rolling motions [*F*_(3, 6)_ = 5.78, *p* = 0.041, $\eta_{\text{P}}^{2}=0.81$] observed in the transfer test (*K* = 5.3) compared to performance on the horizontal-edge route [*K* = 8.4; contrast test: *F*_(1, 8)_ = 10.3, *p* = 0.013, $\eta_{\text{P}}^{2}=0.56$]. Moreover, *neck* rolling motion displayed significantly higher concentration of the distribution [*F*_(3, 6)_ = 6.65, *p* = 0.025, $\eta_{\text{P}}^{2}=0.77$] during the horizontal-edge route [*K* = 12.9; contrast test: *F*_(1, 8)_ = 21.86, *P* = 0.002, $\eta_{\text{P}}^{2}=0.73$] and the vertical-edge route [*K* = 8.1; contrast test: *F*_(1, 8)_ = 7.48, *P* = 0.026, $\eta_{\text{P}}^{2}=0.48$], than in the transfer test (*K* = 5.8). More detailed analyses showed that the transfer test led the climbers to roll their trunk *side* and *obliquely* to the wall for longer durations than for the horizontal-edge and/or vertical-edge and/or double-edge routes (Figures 6, 7). Moreover, the climbers decreased the relative duration spent rolling to the *left side* and increased the relative duration spent rolling to the *right side* during the transfer test in comparison to the other routes (Figures 6, 7).

**Table 3:** **Differences of performatory and exploratory movements, ascent duration and geometric index of entropy between the ***fourth*** learning session of the horizontal-edge, vertical-edge, double-edge routes, and the transfer test**.

	**Performatory movements**			**Exploratory movements**			**Ascent duration (s)**			**Geometric index of entropy**	
	**Mean**	***SD***		**Mean**	***SD***		**Mean**	***SD***		**Mean**	***SD***
Horizontal-edge route	20.7	1.4		0.1	0.3	^*^F_(1, 8)_ = 5.56, *P* = 0.035, $\eta_{\text{p}}^{2}=0.48$	68.8	27.8	^*^F_(1, 8)_ = 12.1, *P* = 0.008, $\eta_{\text{p}}^{2}=0.67$	1.11	0.16
Vertical-edge route	22.2	1.6	^*^F_(1, 8)_ = 10, *P* = 0.013, $\eta_{\text{p}}^{2}=0.56$	0.1	0.3	^*^F_(1, 8)_ = 6.64, *P* = 0.024, $\eta_{\text{p}}^{2}=0.55$	106.8	36.5	^*^F_(1, 8)_ = 10.3, *P* = 0.014, $\eta_{\text{p}}^{2}=0.61$	1.13	0.21
Double-edge route	21.4	1.5		0.2	0.4	^*^F_(1, 8)_ = 5.97, *P* = 0.046, $\eta_{\text{p}}^{2}=0.45$	104.7	26.5	^*^F_(1, 8)_ = 11.2, *P* = 0.010, $\eta_{\text{p}}^{2}=0.65$	1.08	0.13
Transfer test	21.1	1.8		1.7	2.1		140.3	33.3		1.17	0.11
*F*-value	*F*_(3, 6)_ = 5.83			*F*_(1.168, 9.342)_ = 4.38			*F*_(3, 6)_ = 14.41				
*P*-value	*P* = 0.033			*P* = 0.048			*P* = 0.004				
Effect size	$\eta_{\text{p}}^{2}=0.75$			$\eta_{\text{p}}^{2}=0.48$			$\eta_{\text{p}}^{2}=0.88$				

* *Corresponds to significant contrast tests between the fourth learning session of each route and the transfer test with P < 0.05*.

When the behaviors during performance on *unknown routes* (considering the first session of each route) were compared to those observed during the transfer test, we noted higher levels of variability of trunk rolling motion, greater numbers of exploratory movements and longer ascent duration times during the transfer test in comparison to the first session of the horizontal-edge route (Table 4). The higher levels of exploratory behaviors did not decrease climbing fluency because the climbers exhibited statistically non-significant differences in the value of the geometric index of entropy between the first session of the horizontal edge route and the transfer test. These results support the positive effects of exploration during *on-sight climb*ing (notably, on route finding skills as well as movement adaptations). Moreover, although the levels of variability in trunk rolling motions, the number of exploratory movements and the time of ascent did not change significantly between the transfer test and the first session of the double-edge route, performance in the latter showed lower levels of climbing fluency (i.e., higher geometric index of entropy), again supporting the positive effects of movement exploration. More detailed analyses displayed a significant higher concentration of *hip* rolling motion [*F*_(1.395, 11.161)_ = 5.6, *p* = 0.029, $\eta_{\text{P}}^{2}=0.42$] for the horizontal-edge route [*K* = 10.3; contrast test: *F*_(1, 8)_ = 5.68, *p* = 0.044, $\eta_{\text{P}}^{2}=0.42$] than for the transfer test (*K* = 5.3). Moreover, the *neck* rolling motion showed significant higher concentration of the distribution [*F*_(1.306, 10.447)_ = 7.36, *p* = 0.016, $\eta_{\text{P}}^{2}=0.48$] for the horizontal-edge route [*K* = 11.4; contrast test: *F*_(1, 8)_ = 6.56, *p* = 0.034, $\eta_{\text{P}}^{2}=0.45$] than for the transfer test (*K* = 5.8). This can be observed by a longer relative duration of trunk rolling motion *side* and *obliquely* to the wall in the transfer test than in the horizontal-edge climbing condition. This effect was revealed by longer relative duration of time spent in the −90 to −67.5° range of *hip* rolling motion [*F*_(1.675, 13.402)_ = 5.49, *p* = 0.046, $\eta_{\text{P}}^{2}=0.43$] for the transfer test (duration of 4.1 ± 1.8%) than for the horizontal-edge route (duration of 0.2 ± 0.1%) [contrast test: *F*_(1, 8)_ = 5.96, *p* = 0.043, $\eta_{\text{P}}^{2}=0.48$]. Also, longer relative duration of the −90 to −67.5° range of *neck* rolling motion occurred [*F*_(3, 6)_ = 5.41, *P* = 0.044, $\eta_{\text{P}}^{2}=0.73$] for the transfer test (duration of 2.4 ± 1.4%) than for the horizontal-edge route (duration of 0.4 ± 1.1%) [contrast test: *F*_(1, 8)_ = 5.67, *p* = 0.044, $\eta_{\text{P}}^{2}=0.42$].

**Table 4:** **Differences of performatory and exploratory movements, ascent duration and geometric index of entropy between the first learning session of the horizontal-edge, vertical-edge, double-edge routes, and the transfer test**.

	**Performatory movements**		**Exploratory movements**			**Ascent duration (s)**			**Geometric index of entropy**		
	**Mean**	***SD***	**Mean**	***SD***		**Mean**	***SD***		**Mean**	***SD***	
Horizontal-edge route	21.1	0.9	0.2	0.4	^*^F_(1, 8)_ = 5.8, *P* = 0.044, $\eta_{\text{p}}^{2}=0.43$	82.9	31.9	^*^F_(1, 8)_ = 6.77, *P* = 0.031, $\eta_{\text{p}}^{2}=0.46$	1.04	0.10	
Vertical-edge route	22.6	1.1	0.6	0.7		128.0	14.4		1.13	0.18	
Double-edge route	22.8	1.6	0.6	0.7		137.0	39.2		1.33	0.15	^*^F_(1, 8)_ = 11.22, *P* = 0.001, $\eta_{\text{p}}^{2}=0.58$
Transfer test	21.1	1.8	1.7	2.1		140.3	33.3		1.17	0.11	
*F*-value			*F*_(1.567, 12.538)_ = 5.6			*F*_(3, 6)_ = 12.97			*F*_(3, 6)_ = 26.24		
*P*-value			*P* = 0.034			*P* = 0.005			*P* = 0.001		
Effect size			$\eta_{\text{p}}^{2}=0.46$			$\eta_{\text{p}}^{2}=0.87$			$\eta_{\text{p}}^{2}=0.93$		

* *Corresponds to significant contrast tests between the first learning session of each route and the transfer test with P < 0.05*.

## Discussion

### Destabilization and reorganization of a behavioral repertoire

**Figure 8:**
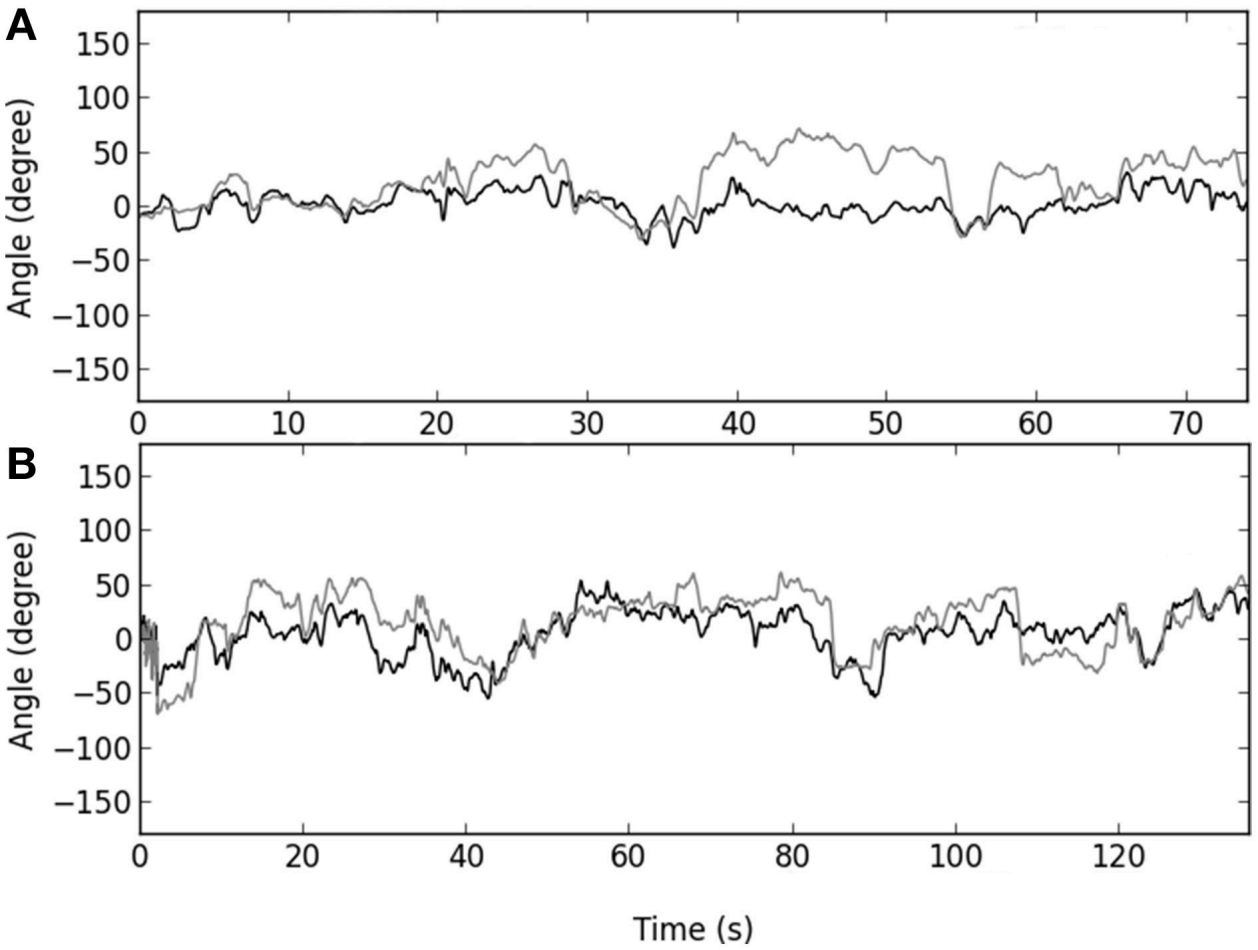
**Angular time-series of the hip (black) and neck (gray) rolling motion for the horizontal-edge route (A) and double-edge route (B) for one individual**. The angular time-series showed higher concentration around μ for the horizontal-edge route (*K* = 22.9 for hip and 9.5 for neck) than for the double-edge route (*K* = 7.7 for hip and 5.2 for neck), meaning that this meta-stable region of performance encourage higher behavioral exploration that could be notably reflected by higher variability of the trunk rolling motion.

Our hypothesis that *route design* manipulation could destabilize a behavioral repertoire was mainly confirmed by the results. Participants completed the climbing task by spending 55–75% of time with a “face to the wall” orientation of the hip and neck whatever the route design, showing the dominance and stability of this pattern in behavioral repertoires. However, there were five main findings that confirmed the hypothesis that this stable state could be destabilized by climbers' emergent behaviors when the route design was manipulated. First, the vertical-edge and the double-edge routes destabilized the range of neck rolling motions that occurred obliquely to the wall 10% of time more often (38 vs. 28%). This emergent behavior is quite understandable because the shoulders are directly linked to the arms and consequently the upper-part of trunk rolling motion is sensitive to the hand orientation during hold grasping. Therefore, oscillating more or less with the trunk may have allowed participants to reach, grasp and use holds that would not be reachable, graspable and usable when a climber attempted to do so with the trunk facing' the wall. Previous work has exemplified that texture, surface, shape, size and orientation of holds invite various hand grasping patterns (e.g., crimp, gaston, jug, mono, pinch, pocket, sloper, and undercling grasping patterns) and body positions (e.g., bridge, campus, crossover, deadpoint, flag, heel hook, knee bar, and mantle body positions; Phillips et al., 2012). These findings support the view that the range of the trunk rolling motion can indicate how a climber is coupled to a performance environment. As suggested in previous studies of motor learning, when an attractor becomes more stable, the stability of the other attractors decrease, causing changes in the entire attractor landscape (behavioral repertoire), shaping an individual's “intrinsic dynamics” (Schöner et al., 1992; Zanone and Kelso, 1992; Zanone et al., 2010). In our study, this reorganization of the trunk rolling motion repertoire was supported by a second important finding: performance on the vertical-edge and the double-edge routes led to a higher level of variability in the neck and hip rolling motion (participants oscillated side-to-side, like a door opening/closing, more often) than on the horizontal-edge route (see Figure 8 for an example of individual angular time-series). Greater levels of local variability in motor organization could reflect enhanced information-movement coupling through better attunement to environmental properties (Fajen et al., 2009).

A previous study in ice climbing has already highlighted the finding that higher local variability of both upper-limbs and lower-limbs in a time-series could characterize higher levels of behavioral adaptation to environmental constraints (Seifert et al., 2013a). For instance, if a climber detects an existing hole in an icefall with his first ice tool swing, he may hook the same hole with the other ice tool to pull upwards (i.e., exhibiting an asymmetric right-left arm coordination), rather than swinging at it when on the same level as the first ice tool (i.e., exhibiting a symmetric right-left arm coordination tendency; Seifert et al., 2014c). These findings suggested how expert ice climbers might exhibit better attunement to environmental properties (e.g., thickness, density, shape, and temperature), which drive and simultaneously are driven by a greater range and higher levels of variability in limb movement and coordination patterns (Seifert et al., 2014c). Our current results confirmed the idea that the reorganization of a pre-existing behavioral repertoire may be interlinked with a better attunement to environmental properties.

Our third finding reinforced this hypothesis by showing that practice on destabilizing route designs, (i.e., vertical-edge and double-edge routes) contributed toward *educating the attention of* learners, as they became more attuned to useful informational variables, i.e., variables *specifying* information for action (Jacobs and Michaels, 2007). Indeed, a greater number of exploratory movements (i.e., touched holds but not grasped) were performed on the vertical-edge and the double-edge routes, supporting the view that environmental constraints influenced the rate of exploration in climbing. This is in line with suggestions of an inverse correlation existing between the average number of grasped holds and the mean velocity of a climber traversing up a vertical surface (Sibella et al., 2007). It was reported that when a climber uses a small number of holds to traverse a climbing wall, being quick enough to maintain equilibrium, whereas when the number of holds used was at least equal to three, a climber tended to exhibit a slower ascent because his/her equilibrium is always under control.

Our last finding related to performance outcome, i.e., the climbing fluency that could be measured by linking time for ascent duration and the geometric index of entropy. Higher values of ascent duration were observed for the vertical-edge and the double-edge routes than on the horizontal-edge route, suggesting more time was spent route finding (i.e., to seek a pathway through multiple choices of holds on a climbing wall) (Cordier et al., 1993, 1994), and/or for exploration of hold grasping actions (for an example of analysis about dual grasping and arm crossing, see Boschker and Bakker, 2002). However, statistically non-significant differences of geometric index of entropy emerged between performance on the horizontal-edge and vertical-edge routes, supporting the view that the destabilization of a behavioral repertoire led to an effective behavioral exploration. One reason for this finding could be the sub-maximal grade of difficulty of this route, which allowed rapid adaptation of climbers to this environmental constraint without too much risk of falling.

### Behavioral exploration during meta-stable region of performance

When greater exploration was encouraged through the design of a meta-stable region of performance, lower climbing fluency emerged on the double-edge route than on the horizontal-edge route, supporting the idea that the level of environmental constraints influenced the level of exploration and performance outcomes in achieving the task-goal (i.e., to climb with the highest level of fluency). However, our results showed that behavioral exploration could also lead to an improvement in climbing fluency because a significant interaction between route design and practice occurred for this double-edge route. This improvement of climbing fluency with practice suggested that the double-edge route, characterized as a “meta-stable region of performance,” not only led to destabilization of a behavioral repertoire, but provided be an opportunity for safe exploration of the perceptual workspace (as already pointed out by Newell et al., 1989, 1991; Newell and McDonald, 1992). Indeed, an easy approach for participants would have been to only perform horizontal hand grasping pattern. Instead, it must be remembered that participants used both vertical (40% of time) and horizontal hand grasping patterns (60% of time) over the four sessions of practice. Therefore, as previously observed by Teulier and Delignières (2007), a meta-stable regime of performance appears to be a safe way to explore various hand grasping patterns as well as trunk rolling motion patterns. Indeed, the double-edge holds offered a fall back strategy toward an already known horizontal hand grasping pattern when the vertical hand grasping pattern appeared unsafe or uncertain. Thus, designing meta-stable regions of performance represents a fruitful condition for exploration where individuals learn to flexibly reorganize their behaviors into a variety of stable patterns of activity (for a study of insect behaviors, see Von Holst, 1973). In our study, we postulated that meta-stable region of performance has invited individuals to continuously adapt and explore, and by doing so they learned to functionally explore the performance environment, i.e., effectively and rapidly. To consider this hypothesis the next section discusses the results of the transfer test, in order to investigate how individuals reinvested their exploratory capabilities in a new context of performance.

### Skill transfer: Individual learnt to functionally explore

Interestingly, individuals exhibited higher levels of exploratory behavior during a transfer test than during sessions of practice. Indeed, exploration was considered as functional and adaptive behaviors because it led to a statistically non-significant difference of the fluency of hip displacement between the transfer test and the fourth session of practice whatever the route. Moreover fluency was greater in the transfer test than in the first session of practice on the double-edge route. The higher level of behavioral exploration was characterized by a higher number of exploratory movements, higher range and variance of trunk rolling motion patterns and in the cases of *unknown routes* by longer ascent duration. These findings suggested that climbers positively reinvested what they learned because they learned how to search and explore functionally, i.e., without deterioration of climbing fluency. Notably, they did not learn a technical solution or a new movement pattern, but they learned to search and explore functionally in order to adapt to environmental constraints (Newell et al., 1989; Newell and McDonald, 1992). In other words, they learnt to be more flexible because they reorganized their entire pre-existing behavioral repertoire, as they were able to roll more obliquely and side to the wall during the transfer test than during the horizontal-edge route.

Secondly, exploratory behavior was more pronounced when performance on a transfer test was compared to performance on *known routes* (i.e., fourth session of practice). Behavior differed with the three other routes; whereas the level of exploration was less marked when the transfer test was compared to *unknown routes* (i.e., first session of practice) because the behavior only differed with the horizontal route, considered as close to pre-existing skills repertoire. This important finding highlights that behavioral exploration is an on-going process that involves continuously both perception and action, but at a different level. According to studies of Cordier et al. (1993, 1994), our results showed that when individuals have to cope with *unknown routes*, they need to find the route to experience which holds are reachable, graspable and usable in order to control their body equilibrium and/or to transit to next hold. Nevertheless, when individuals have to cope with *known routes*, route finding and hold reach-ability are less challenging and exploration mainly concerns hold grasp-ability (i.e., the hand grasping pattern) and use-ability (i.e., the trunk rolling motion which has accompanied the hand grasping pattern). Therefore, the results of the transfer test confirmed that designing a meta-stable region of performance favored functional exploration, because the alternation of exploration (i.e., new hand grasping and rolling motion patterns) and exploitation of pre-existing behavioral pattern allowed “safe” searching strategy. Recently, Pacheco and Newell (2015) confirmed the hypothesis of the effectiveness of a balanced strategy of exploration and stabilization to facilitate transfer in the perceptual-motor workspace. Our study tends to confirm that because meta-stable regime of performance allow fall-back to already stabilized pattern, exploration appears less risky and the learner is more attracted to explore; doing so, he educates his attention by being more attune to specifying information (such as the edge orientation of the hold) for action (Jacobs and Michaels, 2007; Withagen and van der Kamp, 2010). Notably, it must be remembered that the higher number of exploratory movements observed during the transfer test in comparison to the three routes during the fourth session of practice remained only significantly higher in comparison to observations on the horizontal edge route when the first session of practice is taken in account. As this higher number of exploratory movements did not cause any deterioration in performance outcomes (i.e., no change in hip displacement fluency), questions remain over what the learner explores. It is hypothesized that learners explore hold grasp-ability, deciding not to engage the body (exemplified by more or less trunk rolling motion) when the hold did not invite him to a specific grasping pattern. Instead, the learner revises judgment toward another hold or another grasping pattern until being able to use the hold in order to pull-push his body upward. In conclusion, the data reported here suggest that individuals learns to search and explore the perceptual motor workspace during practice (Newell et al., 1991; Newell and McDonald, 1992) in a “safe” way; indeed by designing meta-stable region of performance, we encourage exploration but in the same time, we allow fall-back strategies because the climber can use his pre-existing behavioral repertoire.

### Conflict of interest statement

The authors declare that the research was conducted in the absence of any commercial or financial relationships that could be construed as a potential conflict of interest.

## References

[B1] Amblard B. Assaiante C. Lekhel H. Marchand A. R. (1994). A statistical approach to sensorimotor strategies: conjugate cross-correlations. J. Mot. Behav. 26, 103–112. 10.1080/00222895.1994.9941665 15753063

[B2] Araújo D. Davids K. (2011). What exactly is acquired during skill acquisition? J. Conscious. Stud. 18, 7–23.

[B3] Boschker M. S. J. Bakker F. C. (2002). Inexperienced sport climbers might perceive and utilize new opportunities for action by merely observing a model. Percept. Mot. Skills 95, 3–9. 10.2466/pms.2002.95.1.3 12365268

[B4] Chow J. Y. Davids K. Button C. Rein R. (2008). Dynamics of movement patterning in learning a discrete multiarticular action. Motor Control 12, 219–240. Available online at: http://hdl.handle.net/10497/14246 18698107 10.1123/mcj.12.3.219

[B5] Chow J. Y. Seifert L. Hérault R. Chia S. J. Y. Lee M. C. Y. (2014). A dynamical system perspective to understanding badminton singles game play. Hum. Mov. Sci. 33, 70–84. 10.1016/j.humov.2013.07.016 24075690

[B6] Cohen D. (1988). Statistical Power Analysis for the Behavioral Science, 2nd Edn. Hillsdale, NJ: Erlbaum.

[B7] Cordier P. Dietrich G. Pailhous J. (1996). Harmonic analysis of a complex motor behaviour. Hum. Mov. Sci. 15, 789–807. 10.1016/S0167-9457(96)00023-1

[B8] Cordier P. Mendès-France M. Bolon P. Pailhous J. (1993). Entropy, degrees of freedom, and free climbing: a thermodynamic study of a complex behavior based on trajectory analysis. Int. J. Sport Psychol. 24, 370–378.

[B9] Cordier P. Mendès-France M. Pailhous J. Bolon P. (1994). Entropy as a global variable of the learning process. Hum. Mov. Sci. 13, 745–763. 10.1016/0167-9457(94)90016-7

[B10] Davids K. Araújo D. Hristovski R. Passos P. Chow J. Y. (2012). Ecological dynamics and motor learning design in sport, in Skill Acquisition in Sport: Research, Theory and Practice, eds Hodges N. J. Williams A. M. (New York, NY: Routledge. Taylor and Francis Group), 112–130.

[B11] Davids K. Button C. Bennett S. J. (2008). Dynamics of Skill Acquisition: A Constraints-led Approach, eds Davids K. Button C. Bennett S. J. Champaign, IL: Human Kinetics.

[B12] Delignières D. Famose J. Thépeaut-Mathieu C. Fleurance P. (1993). A psychophysical study of difficulty rating in rock climbing. Int. J. Sport Psychol. 24, 404–416.

[B13] Draper N. Dickson T. Blackwell G. Fryer S. Priestley S. Winter D. (2011). Self-reported ability assessment in rock climbing. J. Sports Sci. 29, 851–858. 10.1080/02640414.2011.565362 21491325

[B14] Fajen B. R. Riley M. R. Turvey M. T. (2009). Information, affordances, and the control of action in sport. Int. J. Sport. Psychol. 40, 79–107. Available online at: http://panda.cogsci.rpi.edu/resources/papers/FajenRileyTurvey2009.pdf

[B15] Faugloire E. Bardy B. G. Stoffregen T. A. (2009). (De)stabilization of required and spontaneous postural dynamics with learning. J. Exp. Psychol. Hum. Percept. Perform. 35, 170–187. 10.1037/0096-1523.35.1.170 19170480

[B16] Gibson J. (1979). The Ecological Approach to Visual Perception. Boston, MA: Houghton Mifflin.

[B17] Howell D. (2002). Statistical Methods for Psychology, 5th Edn. Belmont, CA: Duxbury Press.

[B18] Hristovski R. Davids K. Araújo D. (2006a). Affordance-controlled bifurcations of action patterns in martial arts. Nonlinear Dynamics Psychol. Life Sci. 10, 409–444. 16884651

[B19] Hristovski R. Davids K. Araújo D. (2009). Information for regulating action in sport: metastability and emergence of tactical solutions under ecological constraints, in Perspectives on Cognition and Action in Sport, eds Araujo D. Ripoll H. Raab M. (Hauppauge, NY: Nova Science Publishers), 43–57.

[B20] Hristovski R. Davids K. Araújo D. Button C. (2006b). How boxers decide to punch a target: emergent behaviour in nonlinear dynamical movement systems. J. Sci. Med. Sport 5, 60–73. Available online at: http://www.jssm.org/researchyisi.php?id=jssm-05-CSSI1-60.xml PMC3863932 24357978

[B21] Jacobs D. M. Michaels C. F. (2007). Direct learning. Ecol. Psychol. 19, 321–349. 10.1080/1040741070143233715147322

[B22] Kelso J. A. S. (2008). An essay on understanding the mind. Ecol. Psychol. 20, 180–208. 10.1080/10407410801949297 19865611 PMC2768408

[B23] Kelso J. A. S. (2012). Multistability and metastability: understanding dynamic coordination in the brain. Philos. Trans. R. Soc. Lond. B. Biol. Sci. 367, 906–918. 10.1098/rstb.2011.0351 22371613 PMC3282307

[B24] Kelso J. A. S. Engström D. (2006). The Complementary Nature, eds Kelso J. A. S. Engström D. Cambridge: The MIT Press.

[B25] Kelso J. A. S. Zanone P. G. (2002). Coordination dynamics of learning and transfer across different effector systems. J. Exp. Psychol. Hum. Percept. Perform. 28, 776–797. 10.1037/0096-1523.28.4.776 12190250

[B26] Kostrubiec V. Zanone P.-G. Fuchs A. Kelso J. A. S. (2012). Beyond the blank slate: routes to learning new coordination patterns depend on the intrinsic dynamics of the learner-experimental evidence and theoretical model. Front. Hum. Neurosci. 6:222. 10.3389/fnhum.2012.00222 22876227 PMC3411071

[B27] Ladha C. Hammerla N. Olivier P. Plötz T. (2013). ClimbAX: skill assessment for climbing enthusiasts, in ACM Conference on Ubiquitous Computing, UbiComp'13 Adjunct, eds Häkkila J. Whitehouse K. Krüger A. Tobe Y. Hilliges O. Yatani K. (Zurich: ACM Press), 235–244. 10.1145/2493432.2493492

[B28] Madgwick S. O. H. (2010). An Efficient Orientation Filter for Inertial and Inertial/Magnetic Sensor Arrays. Bristol, UK: University of Bristol Available online at: http://www.x-io.co.uk/res/doc/madgwick_internal_report.pdf

[B29] Madgwick S. O. H. Harrison A. J. L. Vaidyanathan A. (2011). Estimation of IMU and MARG orientation using a gradient descent algorithm. IEEE Int. Conf. Rehabil. Robot. 2011:5975346. 10.1109/icorr.2011.5975346 22275550

[B30] Mardia K. Jupp P. (1999). Directional Statistics. Chichester: John Wiley & Sons, Inc.

[B31] Newell K. M. (1985). Coordination, control and skill, in Differing Perspectives in Motor Learning, eds Goodman D. Wilberg R. B. Franks I. M. (Amsterdam: Elsevier), 295–317.

[B32] Newell K. M. (1986). Constraints on the development of coordination, in Motor Development in Children. Aspects of Coordination and Control, eds Wade M. G. Whiting H. T. A. (Dordrecht: Martinus Nijhoff), 341–360.

[B33] Newell K. M. (1991). Motor skill acquisition. Annu. Rev. Psychol. 42, 213–237. 10.1146/annurev.ps.42.020191.001241 2018394

[B34] Newell K. M. (1996). Change in movement and skill: learning, rentention and transfer, in Dexterity and its Development, eds Latash M. L. Turvey M. T. (Mahwah, NJ: Erlbaum), 393–430.

[B35] Newell K. M. Kugler P. N. Van Emmerik R. E. A. McDonald P. V. (1989). Search strategies and the acquisition of coordination, in Perspectives on the Coordination of Movement, ed Wallace S. A. (Amsterdam: Elsevier), 85–122.

[B36] Newell K. M. McDonald P. V. (1992). Searching for solutions to the coordination function: learning as exploratory behavior. Adv. Psychol. 87, 517–532.

[B37] Newell K. M. McDonald P. V. Kugler P. N. (1991). The perceptual-motor workspace and the acquisition of skill, in Tutorials in Motor Neuroscience, eds Requin J. Stelmach G. E. (Amsterdam: Kluwer Academic), 95–108.

[B38] Newell K. M. Vaillancourt D. E. (2001). Dimensional change in motor learning. Hum. Mov. Sci. 20, 695–715. 10.1016/S0167-9457(01)00073-2 11750683

[B39] Nourrit D. Delignières D. Caillou N. Deschamps T. Lauriot B. (2003). On discontinuities in motor learning: a longitudinal study of complex skill acquisition on a ski-simulator. J. Mot. Behav. 35, 151–170. 10.1080/00222890309602130 12711586

[B40] Pacheco M. Newell K. (2015). Transfer as a function of exploration and stabilization in original practice. Hum. Mov. Sci. 44, 258–269. 10.1016/j.humov.2015.09.009 26415094

[B41] Pansiot J. King R. McIlwraith D. Lo B. Yang G. (2008). ClimBSN: climber performance monitoring with BSN, in IEEE Proceedings of the 5th International Workshop on Wearable and Implantable Body Sensor Networks (Hong Kong: The Chinese University of Hong Kong, IEEE), 33–36. 10.1109/ISSMDBS.2008.4575009

[B42] Phillips K. Sassaman J. Smoliga J. (2012). Optimizing rock climbing performance through sport-specific strength and conditioning. Strength Cond. J. 34, 1–18. 10.1519/SSC.0b013e318255f012

[B43] Pijpers J. R. Oudejans R. D. Bakker F. C. Beek P. J. (2006). The role of anxiety in perceiving and realizing affordances. Ecol. Psychol. 18, 131–161. 10.1207/s15326969eco1803_1

[B44] Pinder R. A. Davids K. Renshaw I. (2012). Metastability and emergent performance of dynamic interceptive actions. J. Sci. Med. Sport 15, 437–443. 10.1016/j.jsams.2012.01.002 22326853

[B45] Reed E. Bril B. (1996). The primacy of action in development. A commentary of N. Bernstein, in Dexterity and its Development, ed Latash M. L. (Hillsdale, NJ: Erlbaum), 431–451.

[B46] Rosalie S. Müller S. (2012). A model for the transfer of perceptual-motor skill learning in human behaviors. Res. Q. Exerc. Sport 83, 413–421. 10.1080/02701367.2012.10599876 22978191

[B47] Sanchez X. Lambert P. Jones G. Llewellyn D. J. (2012). Efficacy of pre-ascent climbing route visual inspection in indoor sport climbing. Scand. J. Med. Sci. Sports 22, 67–72. 10.1111/j.1600-0838.2010.01151.x 20561271

[B48] Schöner G. Zanone P. G. Kelso J. A. S. (1992). Learning as change of coordination dynamics: theory and experiment. J. Mot. Behav. 24, 29–48. 10.1080/00222895.1992.9941599 14766496

[B49] Seifert L. Coeurjolly J. F. Hérault R. Wattebled L. Davids K. (2013a). Temporal dynamics of inter-limb coordination in ice climbing revealed through change-point analysis of the geodesic mean of circular data. J. Appl. Stat. 40, 2317–2331. 10.1080/02664763.2013.810194

[B50] Seifert L. L'Hermette M. Komar J. Orth D. Mell F. Merriaux P. (2014a). Pattern recognition in cyclic and discrete skills performance from inertial measurement units. Procedia Eng. 72, 196–201. 10.1016/j.proeng.2014.06.033

[B51] Seifert L. Orth D. Boulanger J. Dovgalecs V. Hérault R. Davids K. (2014b). Climbing skill and complexity of climbing wall design: assessment of jerk as a novel indicator of performance fluency. J. Appl. Biomech. 30, 619–625. 10.1123/JAB.2014-0052 25010435

[B52] Seifert L. Orth D. Hérault R. Davids K. (2013b). Affordances and grasping patterns variability during rock climbing, in Studies in Perception and Action XII: Seventeenth International Conference on Perception and Action, eds Davis T. Passos P. Dicks M. Weast-Knapp J. (Estoril: Psychology Press, Taylor & Francis), 114–118.

[B53] Seifert L. Wattebled L. Herault R. Poizat G. Adé D. Gal-Petitfaux N.. (2014c). Neurobiological degeneracy and affordance perception support functional intra-individual variability of inter-limb coordination during ice climbing. PLoS ONE 9:e89865. 10.1371/journal.pone.0089865 24587084 PMC3933688

[B54] Seifert L. Wattebled L. L'hermette M. Bideault G. Herault R. Davids K. (2013c). Skill transfer, affordances and dexterity in different climbing environments. Hum. Mov. Sci. 32, 1339–1352. 10.1016/j.proeng.2014.06.033 24055363

[B55] Sibella F. Frosio I. Schena F. Borghese N. A. (2007). 3D analysis of the body center of mass in rock climbing. Hum. Mov. Sci. 26, 841–852. 10.1016/j.humov.2007.05.008 17936389

[B56] Sporns O. Edelman G. M. (1993). Solving Bernstein' s problem: a proposal for the development of coordinated movement by selection. Child Dev. 64, 960–981. 10.2307/1131321 8404271

[B57] Temprado J. J. Della-Grasta M. Farrell M. Laurent M. (1997). A novice-expert comparison of (intra-limb) coordination subserving the volleyball serve. Hum. Mov. Sci. 16, 653–676. 10.1016/S0167-9457(97)00014-6

[B58] Teulier C. Delignières D. (2007). The nature of the transition between novice and skilled coordination during learning to swing. Hum. Mov. Sci. 26, 376–392. 10.1016/j.humov.2007.01.013 17467091

[B59] Tognoli E. Kelso J. A. S. (2009). Brain coordination dynamics: true and false faces of phase synchrony and metastability. Prog. Neurobiol. 87, 31–40. 10.1016/j.pneurobio.2008.09.014 18938209 PMC3020160

[B60] Von Holst E. (1973). The Behavioural Physiology of Animals and Man: The Collected Papers of Erich von Holst. Miami, FL: University of Miami Press.

[B61] Winter E. M. Eston R. G. Lamb K. L. (2001). Statistical analyses in the physiology of exercise and kinanthropometry. J. Sports Sci. 19, 761–775. 10.1080/026404101317015429 11561673

[B62] Withagen R. de Poel H. J. Araújo D. Pepping G.-J. (2012). Affordances can invite behavior: reconsidering the relationship between affordances and agency. New Ideas Psychol. 30, 250–258. 10.1016/j.newideapsych.2011.12.003

[B63] Withagen R. van der Kamp J. (2010). Towards a new ecological conception of perceptual information: lessons from a developmental systems perspective. Hum. Mov. Sci. 29, 149–163. 10.1016/j.humov.2009.09.003 20061040

[B64] Zanone P. G. Kelso J. A. S. (1992). Evolution of behavioral attractors with learning: nonequilibrium phase transition. J. Exp. Psychol. Hum. Percept. Perform. 18, 403–421. 10.1037/0096-1523.18.2.403 1593227

[B65] Zanone P. G. Kelso J. A. S. (1997). Coordination dynamics of learning and transfer: collective and component levels. J. Exp. Psychol. Hum. Percept. Perform. 23, 1454–1480. 10.1037/0096-1523.23.5.1454 9336961

[B66] Zanone P. G. Kostrubiec V. Alabaret J. M. Temprado J. J. (2010). Covariation of attentional cost and stability provides further evidence for two routes to learning new coordination patterns. Acta Psychol. (Amst). 133, 107–118. 10.1016/j.actpsy.2009.10.006 19939341

